# Factors associated with improvement in symptoms and quality of life for first-line EGFR-tyrosine kinase inhibitor treatment in patients with *EGFR*-mutated non-small-cell lung cancer - A multicenter prospective SMILE study

**DOI:** 10.7150/jca.30507

**Published:** 2019-07-10

**Authors:** Yu-Feng Wei, Wen-Tsung Huang, Tu-Chen Liu, Jiunn-Min Shieh, Chih-Feng Chian, Ming-Fang Wu, Chih-Cheng Chang, Ching-Hsiung Lin, Jen-Chung Ko, Chia-Mo Lin, Te-Chun Hsia

**Affiliations:** 1 Division of Chest Department, Department of Internal Medicine, E-Da Hospital, I-Shou University, Kaohsiung, Taiwan.; 2 Institute of Biotechnology and Chemical Engineering, I-Shou University, Kaohsiung, Taiwan; 3 Division of Hematology-Oncology, Department of Internal Medicine, Chi Mei Medical Center Liouying, Tainan, Taiwan; 4 Department of Chest Medicine, Cheng Ching Hospital-Chung Kang Branch, Taichung, Taiwan; 5 Department of Internal Medicine, Chi Mei Medical Center - YongKang Branch, Tainan, Taiwan; 6 Division of Pulmonary and Critical Care Medicine, Internal Medicine Department, Tri‐Service General Hospital, National Defense Medical Center, Taipei, Taiwan; 7 Division of Medical Oncology, Department of Internal Medicine, Chung Shan Medical University Hospital, and School of Medicine, Chung Shan Medical University, Taichung, Taiwan; 8 Division of Pulmonary Medicine, Department of Internal Medicine, Shuang Ho Hospital, Taipei, Taiwan; 9 Department of Internal Medicine, School of Medicine, College of Medicine, Taipei Medical University.; 10 Division of Chest Medicine, Department of Internal Medicine, Changhua Christian Hospital, Changhua, Taiwan; 11 Department of Internal Medicine, National Taiwan University Hospital - Hsinchu Branch, Hsinchu, Taiwan; 12 Sleep Center, Pulmonary and Critical Care Medicine, Shin Kong Wu Ho‐Su Memorial Hospital, Taipei, Taiwan; 13 Department of Respiratory Therapy, China Medical University, Taichung, Taiwan; 14 Division of Pulmonary and Critical Care Medicine, Department of Internal Medicine, China Medical University Hospital, China Medical University, Taichung, Taiwan

**Keywords:** epidermal growth factor receptor tyrosine kinase inhibitor, non-small cell lung cancer, quality of life

## Abstract

**Introduction**: Epidermal growth factor receptor-tyrosine kinase inhibitors (EGFR-TKIs) are a standard first-line treatment for advanced *EGFR*-mutated NSCLC patients. Factors associated with symptoms and quality of life (QOL) improvements have not been investigated.

**Methods**: We conducted a multicenter, prospective study to evaluate improvements in QOL and symptoms in NSCLC patients treated with first-line EGFR-TKIs. QOL was assessed using the instrument of Functional Assessment of Cancer Therapy-Lung questionnaire (FACT-L) and Treatment Outcome Index (TOI). Assessment of symptoms was evaluated using the Lung cancer subscale (LCS).

**Results**: Eligible subjects included 280 patients for endpoint analyses. The mean FACT-L score increased by 4.0 ± 15.56 at Week 2 (p<0.001), 5.1 ± 18.48 at Week 4 (p<0.001), and 4.2 ± 20.27 at Week 12 (p=0.001). Similarly, a 2.3 ± 11.65 (p<0.001), 3.2 ± 13.59 (p<0.001), and 2.4 ± 14.34 (p=0.009) increase in mean TOI score were observed at Weeks 2, 4 and 12, respectively. For LCS, it was slightly increased by 1.7 ± 4.61, 2.0 ± 5.50, and 2.0 ± 5.36 at Weeks 2, 4, and 12 (all p<0.001), respectively. Subgroup analyses showed patients who were ex-smokers or with at least 3 metastatic sites were associated with symptoms improvement. Patients who were ex-smokers, with at least 3 metastatic sites, a PS of 1, or treated with gefitinib were associated with QOL improvement.

**Conclusions**: In *EGFR* -mutated NSCLC patients who were treated with first-line EGFR-TKIs, these ex-smokers or with 3 or more metastatic sites were associated with improvements in symptoms and QOL.

## Introduction

Lung cancer is the leading cause of cancer deaths around the world, including Taiwan.^1^ Non-small cell lung cancer (NSCLC) accounts for more than 85% of all lung cancer cases and more than 70% of lung cancer patients present with advanced disease (stage III or IV) at initial diagnosis.^2^

NSCLC patients with *EGFR* mutation(s) have been observed to have a higher response rate, longer progression-free survival and better tolerability to EGFR-tyrosine kinase inhibitors (EGFR-TKIs) compared with standard first-line platinum-based doublet chemotherapy.^3^-^5^ In addition to prolonged progression-free survival, controlling the symptoms and improving the quality of life (QOL) of patients are also key goals in the treatment of advanced NSCLC. Post-hoc analyses of large randomized trials demonstrated EGFR-TKIs, including gefitinib, erlotinib, and afatinib, were associated with better symptom control and QOL outcomes in *EGFR* -mutated NSCLC patients when compared to conventional chemotherapy.^6^-^9^ In the LUX-Lung 7 trial, similar improvements in patient-reported outcomes and QOL were reported in patients treated with afatinib and gefitinib.^10^ To the best of our knowledge, however, factors associated with improvements in symptoms and QOL with EGFR-TKI treatment have not been investigated. The aim of this study was to examine the extent patient characteristics have on the improvement of symptoms and QOL in a real-world patient population in Taiwan treated with EGFR-TKIs.

## Materials and Methods

### Patient selection criteria and study design

An open-label, non-interventional, single-arm, multicenter prospective observational study was conducted across 7 medical centers and 5 regional hospitals in Taiwan. Patients who fulfilled all of the following criteria were eligible for the study: **1)** provision of informed consent form, **2)** aged 20 years and older, **3)** diagnosed with locally advanced or metastatic (stage IIIb/IV) NSCLC and confirmed positive for *EGFR* mutation, **4)** treatment-naïve and ready for the prescription of a EGFR-TKI as their first-line cancer treatment, **5)** able to complete the questionnaires. Patients were excluded from the study if they were involved in the planning and/or the progress of the clinical trials. The study was reviewed and approved by all the Institutional Review Board of the participating institutes.

EGFR-TKI therapy of 250 mg gefitinib (Iressa^®^, AstraZeneca, Cambridge, England), 150 mg erlotinib (Tarceva^®^, Hoffmann-La Roche, Basel Switzerland) or 40 mg afatinib (Giotrif^®^, Boehringer Ingelheim, Ingelheim, Germany) was prescribed to patients by physicians at baseline according to physicians' judgment under the real-world settings. Drugs were administrated daily by investigators and no patients changed medications during the course of the study.

### Data collection and measurements for symptoms and QOL

Demographic and clinical data related to lung cancer were collected, including age, gender, smoking status (number and duration), staging at diagnosis, metastatic site, subtype of *EGFR* mutation, performance status (PS) and concomitant diseases at baseline. QOL was assessed using the Functional Assessment of Cancer Therapy-Lung (FACT-L) questionnaire and Treatment Outcome Index (TOI) derived from FACT-L. Improvement to QOL was defined as an increase in FACT-L or TOI score by ≥ 6 points of change from baseline.^11^ Improvement in disease related symptoms was measured by the Lung Cancer Subscales (LCS) of FACT-L questionnaire. A clinically meaningful improvement in symptoms was defined as an increase in LCS by ≥ 2 points from baseline.^12^ An LCS increase/decrease of ≤ 2 points from baseline was defined as no change. Worsening in symptoms was defined as a decrease in LCS by ≥ 2 points from baseline.^12^ The instrument was completed at pre-treatment (baseline), every 2 weeks in the first month, and 3 months after administration of EGFR-TKIs.

### Statistical analysis

Data are presented as mean ± SD (or SE) for continuous parameters, and as number and percentage for categorical parameters. To evaluate improvement in symptoms, the evaluable-for-symptom improvement (EFS) population was used for data analysis, which was defined as patients with an evaluable baseline LCS assessment and at least one evaluable post-baseline assessment. The mean change in FACT-L, TOI, and LCS scores at Weeks 2, 4, and 12 from baseline was analyzed within the EFS population using the paired sample t-test. For subgroup analyses, the number and proportion of patients who achieved improvement during the study period was calculated by each subgroup with an exact 95% confidence interval, to clarify the factors associated with the improvement in symptoms and QOL in patients treated with EGFR-TKI.

All tests for significance were two-sided, and a p-value of less than 0.05 was considered to be statistically significant. All analyses were performed using Statistical Analysis System® (SAS) for Windows (Version 9.3 or higher, SAS Institute, Cary, North Carolina, USA) statistical package.

## Results

Between November 7^th^ 2013 and June 30^th^ 2015, a total of 346 patients with NSCLC were screened for study entry. 292 patients (84.4%, 292/346) met all screening criteria and were enrolled in this study. Of these patients, 280 were included in the EFS population for endpoint analyses. Demographic and characteristics of the EFS population are presented in **Table 1.** The average age was 65.3 ± 12.38 years old, ranging from 31.1 to 90.4 years. A large proportion of the EFS population was female (63.2%) and there was a greater proportion of never-smokers (73.9%) than former and current-smokers. Most patients had a PS score of 0-1 (83.9%). The major subtypes of *EGFR* mutation were exon 21 L858R (50.3%) and exon 19 (44.3%) deletion. For treatment, 72.1%, 19.3% and 8.6% of patients were treated with gefitinib 250 mg erlotinib 150 mg and afatinib 40 mg, respectively. **Table 2** summarizes the change in symptoms (LCS) and QOL (FACT-L or TOI) scores at Weeks 2, 4, and 12 from baseline following EGFR-TKI therapy. 45.7% of patients achieved clinically meaningful improvement in symptoms (LCS ≥ 2 points change from baseline) at Week 2 and this proportion was sustained to Week 4 (43.6%) and Week 12 (44.6%). The average LCS score was observed to slightly increase over baseline measurements by 1.7 ± 0.28 at Week 2, 2.0 ± 0.33 at Week 4, and 2.0 ± 0.34 at Week 12 (all p<0.001). The average change in mean LCS at Weeks 4 and 12 were clinically meaningful.

In regards to improvement to QOL, the mean FACT-L score was observed to increase by 4.0 ± 0.93 at Week 2, 5.1 ± 1.12 at Week 4, and 4.2 ± 1.28 at Week 12 (all p<0.001). Similarly, an increase in mean TOI score of 2.3 ± 0.70 (p<0.001), 3.2 ± 0.82 (p<0.001), and 2.4 ± 0.91 (p=0.009) were observed at Weeks 2, 4, and 12, respectively. In contrast to the meaningful change observed in LCS score, changes in mean FACT-L and TOI score were mostly not clinically meaningful.

**Figure 1:**
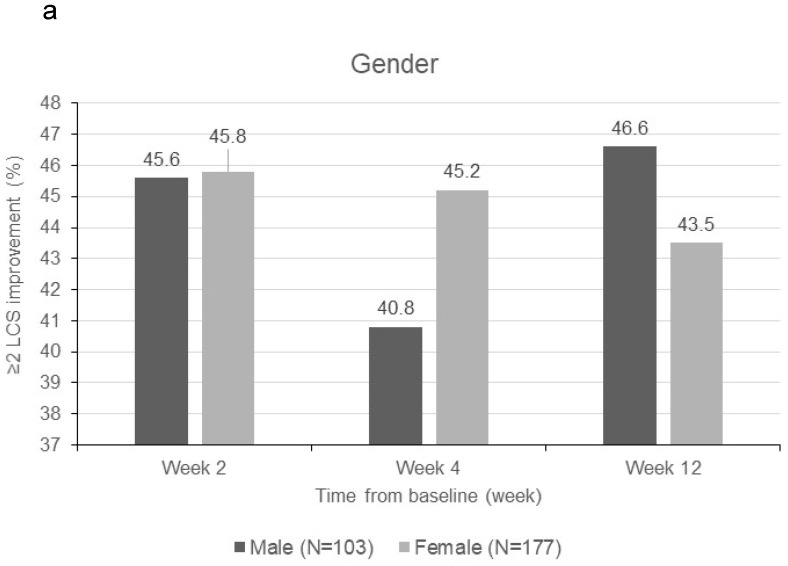

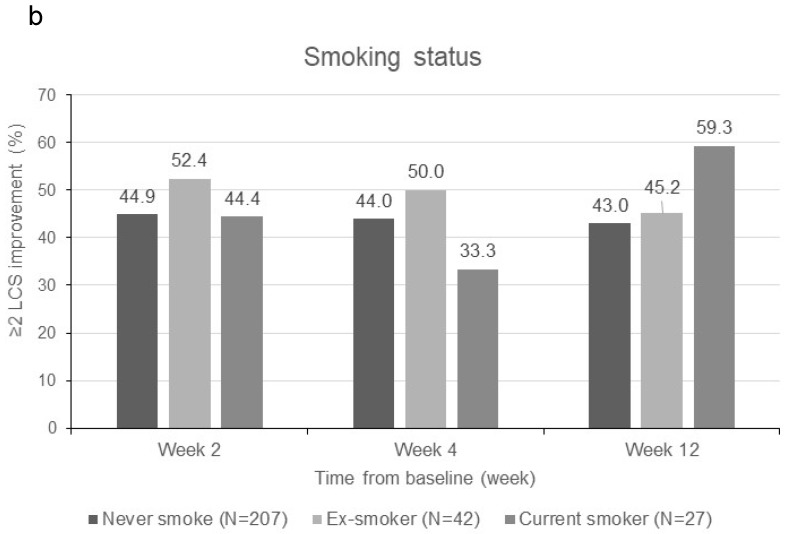

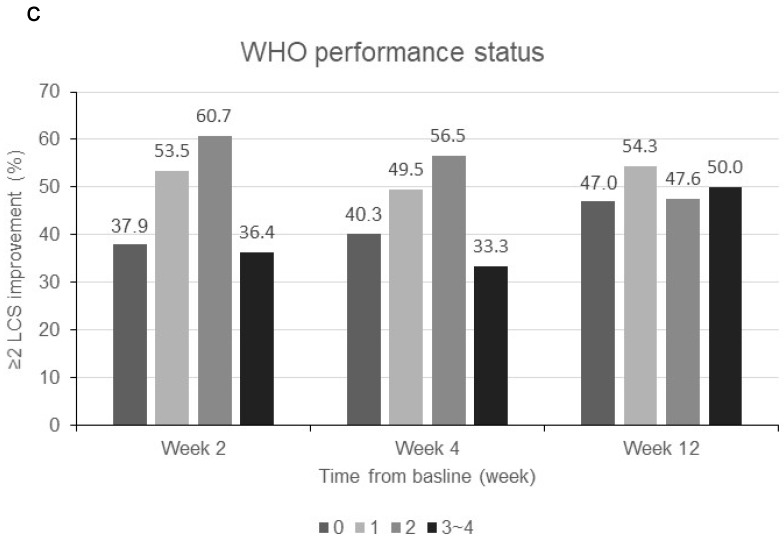

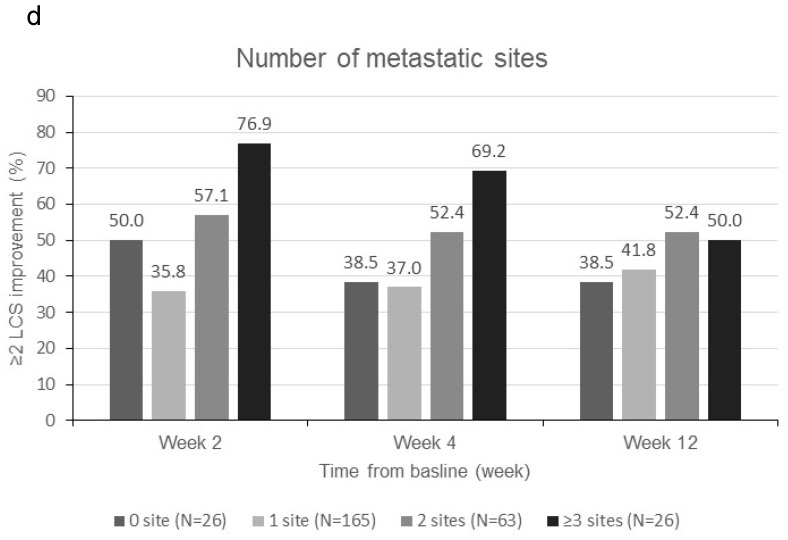

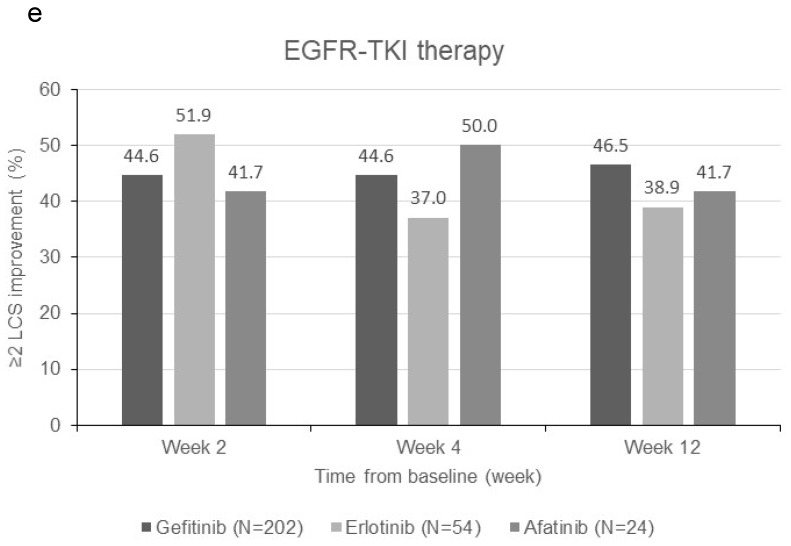
Subgroup analyses for clinically meaningful improvement (LCS ≧ 2 points from baseline score). **a)** gender; **b)** smoking status; **c)** WHO performance status, **d)** number of metastatic sites and **e)** EGFR-TKI therapy. LCS = Lung cancer subscale; EGFR-TKI = epidermal growth factor receptor-tyrosine kinase inhibitor

**Figure 2:**
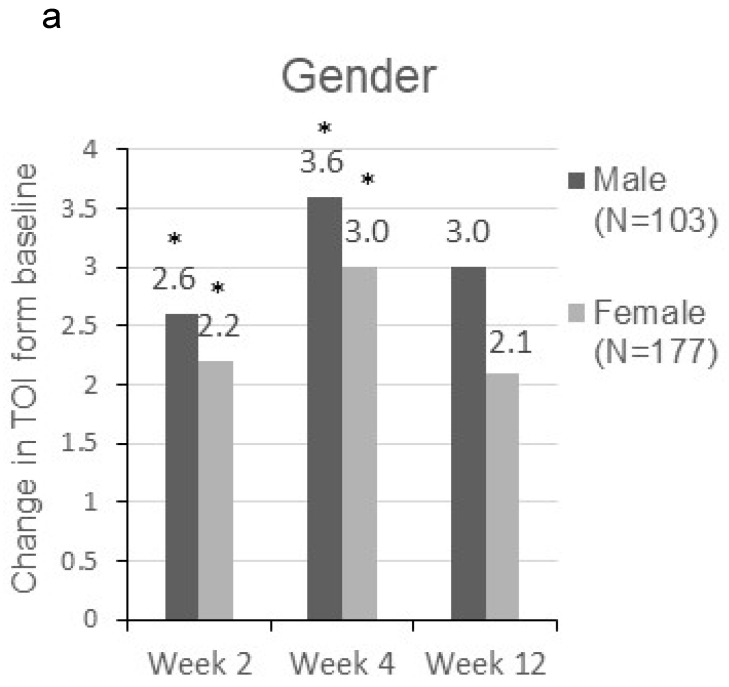

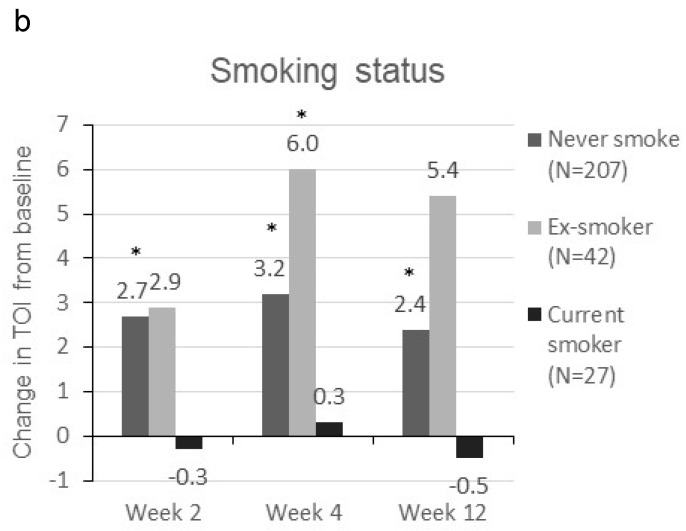

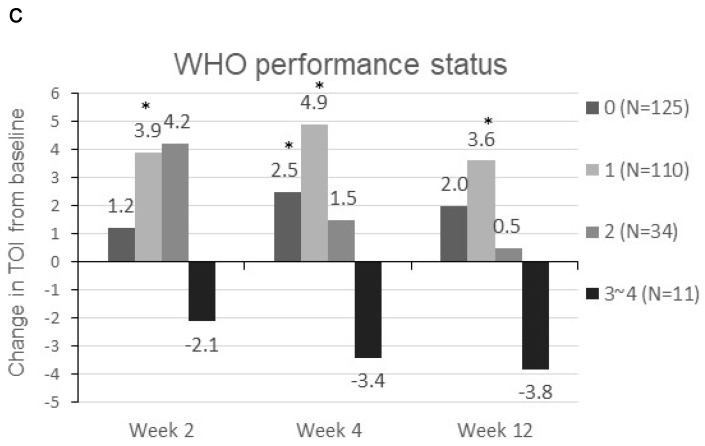

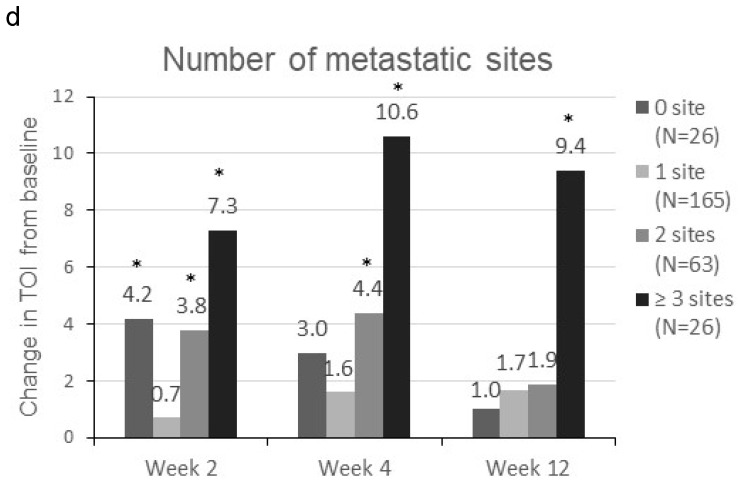

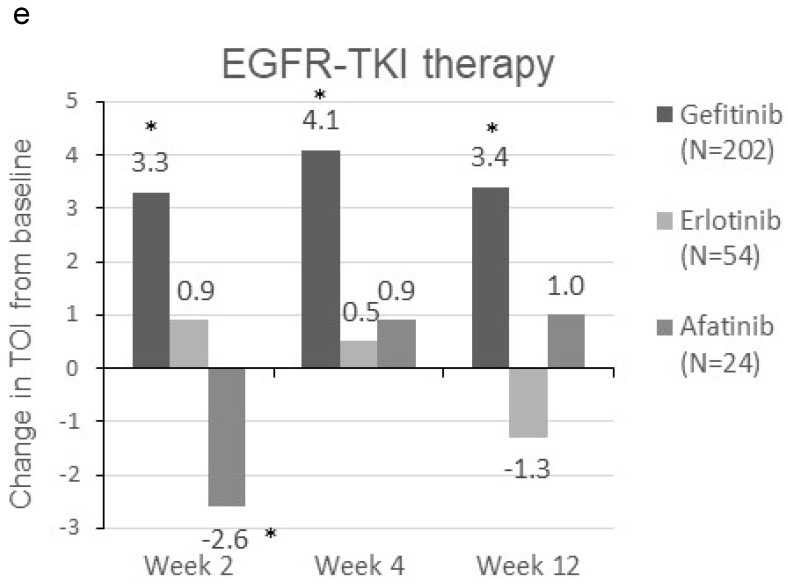

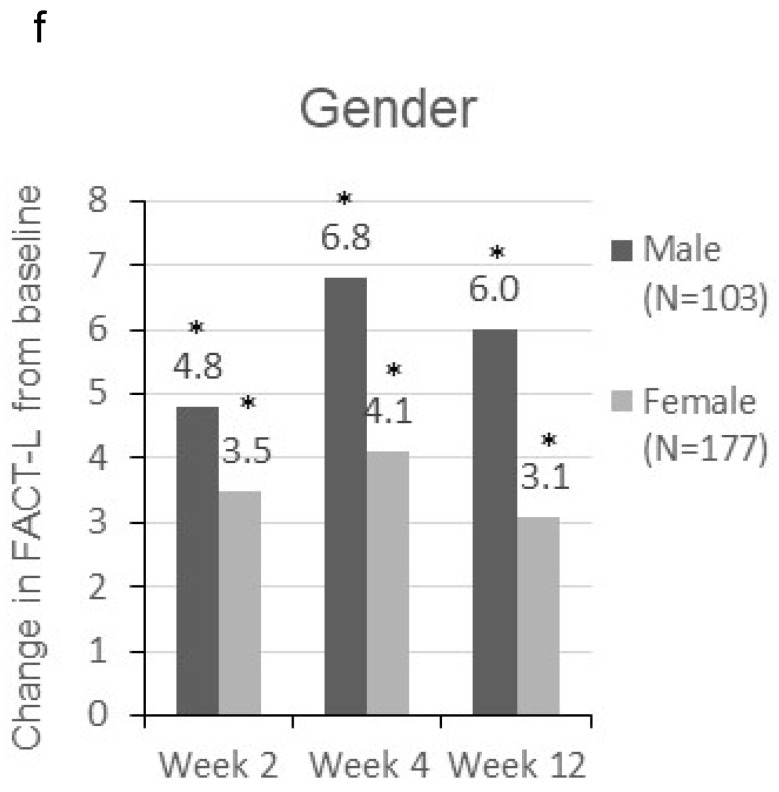

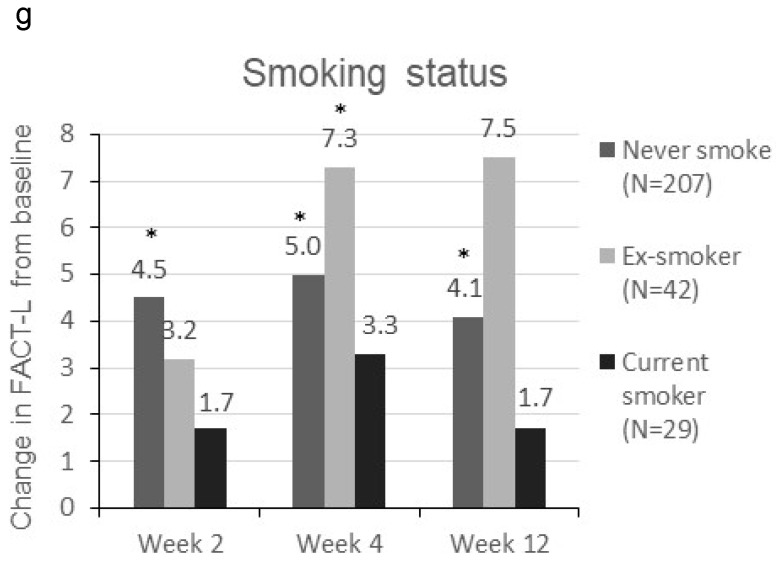

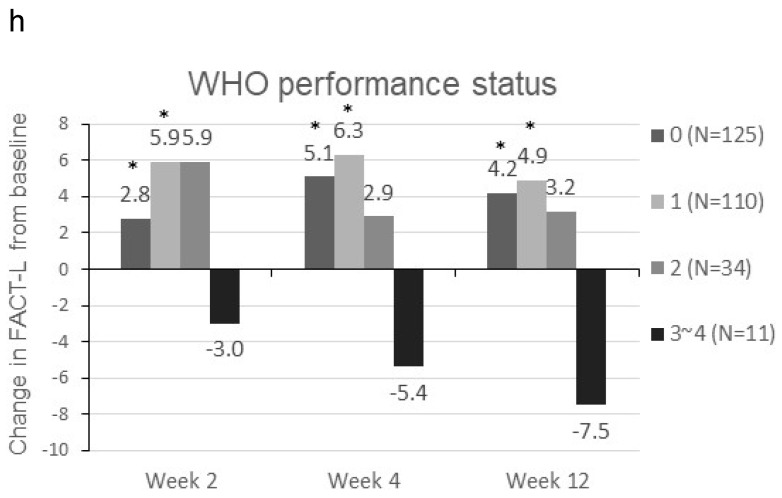

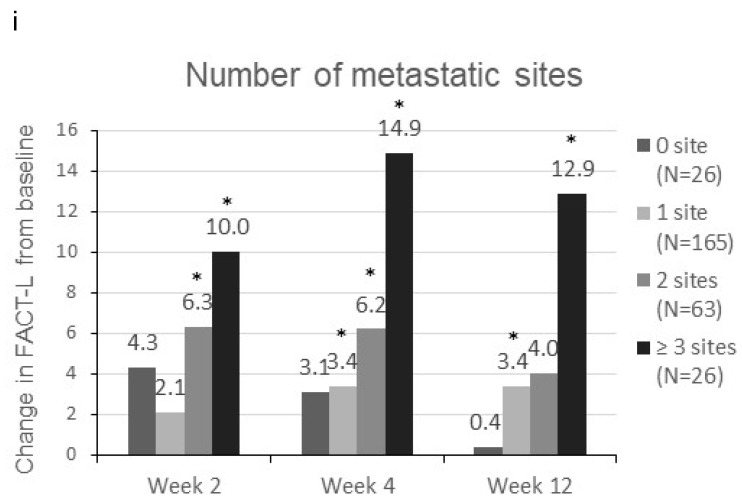

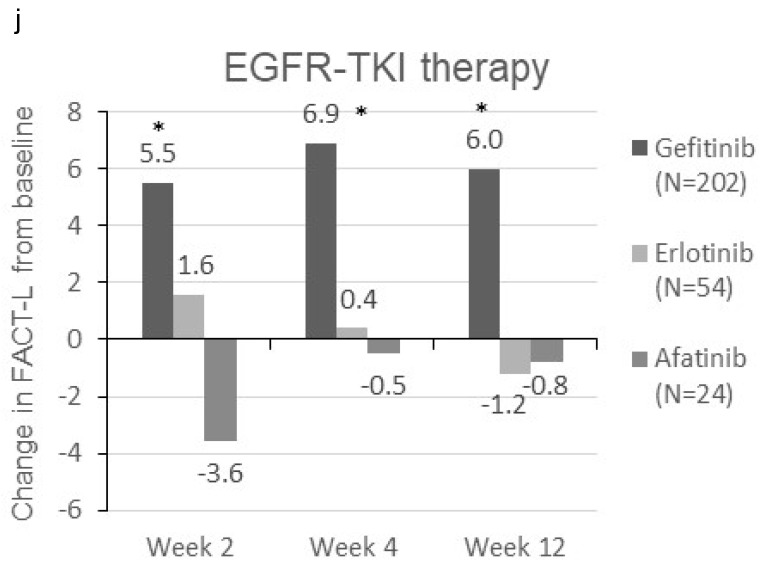
Change in TOI **(a-e)** and FACT-L **(f-j)** from baseline by subgroups. FACT-L = Functional Assessment of Cancer Therapy-Lung questionnaire; TOI = Treatment Outcome Index. * Score changes from baseline with p-value < 0.05

Patients were sorted by gender, smoking status, PS, number of metastatic sites and EGFR-TKI therapy for subgroup analyses. Clinically meaningful improvement of symptoms is presented in **Figure 1**. Patients who were ex-smokers or with at least 3 metastatic sites were associated with increased symptoms improvement in terms of LCS. In addition, patients who are ex-smokers, with at least 3 metastatic sites, a PS of 1 or treated with gefitinib were generally associated with improved QOL in terms of TOI and FACT-L (**Figure 2**).

## Discussion

In this prospective observational study, we provided a comprehensive assessment of symptom burden and QOL in patients with advanced NSCLC based on diverse patient-reported outcomes evaluated with FACT-L, LCS, and TOI in Taiwan. Our study indicated that patients with advanced *EGFR*-mutated NSCLC displayed an improvement in symptoms and QOL after the introduction of EGFR-TKI therapy. Although the overall increments of TOI and FACT-L did not reach defined clinically meaningful improvement (change in TOI or FACT-L ≥ 6 points from baseline score), the average change in LCS at week 4 and week 12 were clinically meaningful (≥ 2 points change from baseline). In addition, all changes in TOI, FACT-L, and LCS of EFS population were statistically significant (p<0.05). In general, the result may provide insights for clinical care.

EGFR-TKI therapy was found to be associated with a higher response rate in patients with advanced *EGFR*-mutated NSCLC.^3^-^5^ Pooled data from the LUX-Lung 3 and LUX-Lung 6 trials demonstrated a survival benefit in patients with exon 19 deletion *EGFR* mutations.^3^ However, previous randomized trials with gefitinib and erlotinib have not shown that these patients have an increased survival advantage.^4^, ^5^ Nevertheless, it has been demonstrated that first-line EGFR-TKIs offer an advantage compared with chemotherapy in symptom control and QOL improvements for patients with advanced *EGFR*-mutated NSCLC.^6^-^9^

In addition to patient-reported outcome assessments, the present study also provides an insight into the association of different patient characteristics with improvements in symptoms and QOL. For subgroup analyses, patients were sorted by gender, smoking status, PS, number of metastatic sites or EGFR-TKI therapy. Previous studies have shown brain and bone metastases are associated with a generally poor survival outcome and low QOL.^13^, ^14^ Oh et al. reported tumor burden and the number of metastatic sites are predictors of poor outcomes in patients with NSCLC.^15^

Smoking is another important factor associated with the worsening of symptoms and poorer QOL on diagnosis and after treatment.^16^, ^17^ EGFR-TKIs have been demonstrated to have activity in *EGFR*-mutated patients with brain metastasis with a response rate of 70-80%.^18^ In addition, *EGFR* signaling is an important mediator of bone metastasis in many cancers. EGFR-TKIs may block osteoclast activation and enhance osteoblastic reactions in those patients.^19^, ^20^ Our study indicated patients that were ex-smokers or patients with at least 3 metastatic sites, probably had worse symptoms and lower QOL at baseline and were associated with increased symptoms and QOL improvement after the introduction of EGFR-TKI treatment.

Our study also showed that patients with a PS of 1 and patients that received gefitinib treatment were also associated with improved QOL. Previous studies have indicated gefitinib and erlotinib may provide clinical benefits to *EGFR*-mutated patients with poor PS (≤2).^21^-^24^ Inoue and colleagues has previously reported the outcome of gefitinib treatment in 30 NSCLC patients with mutated *EGFR* and poor PS (≤2); including 22 patients with a PS of 3-4. The overall response rate to first-line gefitinib treatment was 66% and the disease control rate was 90%. The rate of PS improvement was 79% and 68% of the 22 patients improved from a PS of 3-4 at baseline to a PS of 0-1 at the conclusion of the study.^23^ However, a good PS at diagnosis is associated with better clinical outcome in those patients.^24^, ^25^

Diarrhea and skin rashes are the most frequent adverse effects related with EGFR-TKI toxicity. These adverse symptoms are observed to occur in more than half of NSCLC patients with *EGFR* mutation(s).^26^, ^27^ A meta-analysis reported gefitinib was associated with lower treatment-related diarrhea and skin rash.^26^ Lower treatment-related toxicities could substantially improve QOL, which was consistent with our finding in this study.

Osimertinib is a third-generation EGFR-TKI which has been approved and may now be used as a first-line treatment for advanced EGFR-mutated NSCLC patients. Treatment with osimertinib in the FLAURA trial showed significantly improved progression-free survival compared to patients on Gefitinib or Erlotinib, with a lower rate of serious adverse events.^28^ However, Osimertinib may not available or affordable in certain countries. Gefitinib probably a preferred option as first-line treatment in those patients from the perspective of symptoms and QOL improvements.

A limitation of our study was subject to a real-world, population-based setting, the imbalanced population sorted according to different patient characteristics was inevitable. For example, most of the patients included in the study were treated with gefitinib, which was the first approved and launched EGFR-TKI in Taiwan. Relatively few patients were treated with erlotinib or afatinib in this study. In addition, the sample size was relatively small which may induce a cases bias and limit the possibility for general implications. A large-scale study is suggested for future research to collect more real-world data to confirm the trends observed in the present study.

## Conclusion

Our findings suggest that symptom burden and QOL were slightly improved in *EGFR*-mutated advanced NSCLC patients treated with EGFR-TKIs as first-line therapy. Subgroup analyses showed that patients that were ex-smokers or with 3 or more metastatic sites were associated with improvements in symptoms and QOL. Moreover, patients with a PS of 1 or treated with gefitinib were also associated with improvement in QOL. These results may provide insights for clinical care in patients treated with EGFR-TKIs.

## Figures and Tables

**Table 1:** Demographic information and baseline characteristics of patients.

Characteristics		EFS population, N=280
**Age (year)**	Mean ± SD	65.3 ± 12.38
**Gender**	Male	103(36.8)
	Female	177(63.2)
**Smoking Status**	Never Smoke	207(73.9)
	Ex-smoker	42(15.0)
	Current smoker	27(9.6)
	Occasionally smoke	3(1.1)
	Missing	1(0.4)
**Staging at enrollment**	IIIB	26(9.3)
	IV	254(90.7)
**Metastatic sites at enrollment**	Bone	106(37.9)
	Lung	102(36.4)
	Brain	58(20.7)
	None	28(10.0)
	Liver	23(8.2)
	Adrenal	6(2.1)
	Other	78(27.9)
***EGFR* mutation^#^**	Common mutation*	265(94.6)
	Uncommon mutation	15(5.4)
**EGFR-TKI therapy**	Gefitinib	202 (72.1)
	Erlotinib	54(19.3)
	Afatinib	24(8.6)
**WHO performance status**	0	125(44.6)
	1	110(39.3)
	2	34(12.1)
	3-4	11(3.9)

Data were presented as N (%) *Common *EGFR* mutations are defined as mutations in exon 19 or 21; uncommon mutations are defined as mutations in exon 18 or exon 20 EFS = evaluable-for-symptom improvement

**Table 2:** Changes in symptoms (LCS) and QOL (FACT-L and TOI) response of EFS population following EGFR-TKI therapy.

Summary of response		Week 2	Week 4	Week 12
**Changes from baseline by visits**	EFS population	280	270	251
**Response^a^**	Improvement	128(45.7)	122(43.6)	125(44.6)
	Stable/No change	91(32.5)	99(35.4)	88(31.4)
	Worsening	61(21.8)	59(21.1)	67(23.9)
**Changes in LCS**	Mean ± SE	1.7±0.28	2.0±0.33	2.0±0.34
	p-value	<0.001	<0.001	<0.001
**Changes in FACT-L^b^**	Mean ± SE	4.0±0.93	5.1±1.12	4.2±1.28
	p-value	<0.001	<0.001	0.001
**Changes in TOI^c^**	Mean ± SE	2.3±0.70	3.2±0.82	2.4±0.91
	p-value	<0.001	<0.001	0.009

^a^ Improvement is defined as an increase in LCS ≥ 2 points; worsening is defined as a decrease in LCS ≥ 2 points; stable/no change is defined as a change in LCS between ‐2 and 2 points. Data were presented as N (%) ^b^ Change in TOI ≥ 6 points from baseline score indicates a clinically relevant improvement to QOL. ^c^ Change in FACT-L ≥6 points from baseline score indicates a clinically relevant improvement to QOL. EFS = evaluable-for-symptom improvement; LCS = Lung cancer subscale; FACT-L = Functional Assessment of Cancer Therapy-Lung questionnaire; TOI = Treatment Outcome Index
